# The formyl peptide receptor like-1 and scavenger receptor MARCO are involved in glial cell activation in bacterial meningitis

**DOI:** 10.1186/1742-2094-8-11

**Published:** 2011-02-07

**Authors:** Benedikt J Braun, Alexander Slowik, Stephen L Leib, Ralph Lucius, Deike Varoga, Christoph J Wruck, Sandra Jansen, Rainer Podschun, Thomas Pufe, Lars-Ove Brandenburg

**Affiliations:** 1 Department of Anatomy and Cell Biology, RWTH Aachen University, Germany; 2 Institute for Infectious Diseases, University of Bern, Switzerland; 3 Department of Anatomy, Christian-Albrechts-University Kiel, Germany; 4 Department of Trauma, University Hospital Schleswig-Holstein, Campus Kiel, Germany; 5 Institute of Infection Medicine, University Hospital Schleswig-Holstein, Campus Kiel, Germany

## Abstract

**Background:**

Recent studies have suggested that the scavenger receptor MARCO (macrophage receptor with collagenous structure) mediates activation of the immune response in bacterial infection of the central nervous system (CNS). The chemotactic G-protein-coupled receptor (GPCR) formyl-peptide-receptor like-1 (FPRL1) plays an essential role in the inflammatory responses of host defence mechanisms and neurodegenerative disorders such as Alzheimer's disease (AD). Expression of the antimicrobial peptide cathelicidin CRAMP/LL-37 is up-regulated in bacterial meningitis, but the mechanisms underlying CRAMP expression are far from clear.

**Methods:**

Using a rat meningitis model, we investigated the influence of MARCO and FPRL1 on rCRAMP (rat cathelin-related antimicrobial peptide) expression after infection with bacterial supernatants of *Streptococcus pneumoniae *(SP) and *Neisseria meningitides *(NM). Expression of FPRL1 and MARCO was analyzed by immunofluorescence and real-time RT-PCR in a rat meningitis model. Furthermore, we examined the receptor involvement by real-time RT-PCR, extracellular-signal regulated kinases 1/2 (ERK1/2) phosphorylation and cAMP level measurement in glial cells (astrocytes and microglia) and transfected HEK293 cells using receptor deactivation by antagonists. Receptors were inhibited by small interference RNA and the consequences in NM- and SP-induced Camp (rCRAMP gene) expression and signal transduction were determined.

**Results:**

We show an NM-induced increase of MARCO expression by immunofluorescence and real-time RT-PCR in glial and meningeal cells. Receptor deactivation by antagonists and small interfering RNA (siRNA) verified the importance of FPRL1 and MARCO for NM- and SP-induced Camp and interleukin-1β expression in glial cells. Furthermore, we demonstrated a functional interaction between FPRL1 and MARCO in NM-induced signalling by real-time RT-PCR, ERK1/2 phosphorylation and cAMP level measurement and show differences between NM- or SP-induced signal transduction.

**Conclusions:**

We propose that NM and SP induce glial cell activation and rCRAMP expression also via FPRL1 and MARCO. Thus the receptors contribute an important part to the host defence against infection.

## Background

Acute meningitis, caused by microorganisms such as bacteria, viruses, fungi and parasites, is a severe inflammatory CNS disease. Bacterial meningitis alone accounts for approximately 171,000 annual deaths worldwide (WHO Health Report 2000). As the leading pathogens of bacterial meningitis, *Streptococcus pneumoniae *and *Neisseria meningitidis *comprise almost 80% of all cases [1]. Aside from the blood-brain barrier, innate immunity forms the first line of defence for pneumococci and meningococci [2]. Host cells of the central nervous system, especially microglia and astrocytes, release many factors, which help to manage the infection and coordinate host defence cells [3]. Amongst these factors are also antimicrobial peptides. Antimicrobial peptides are effector molecules of the innate immune system that have microbiocidal and proinflammatory properties [4,5]. Our previous results show an increase of antimicrobial peptides (for example human cathelicidin LL-37) in CSF of patients with bacterial meningitis but not in control CSF [6]. Our results also revealed increased expression of the rat cathelin-related antimicrobial peptide (rCRAMP) by astrocytes and by microglia as well as meningeal cells *in vivo *and *in vitro *after bacterial stimulation [6,7]. Cathelicidins are defined by a highly conserved N-terminal cathelin pro-domain and a structurally variable antimicrobial domain at the C terminus [8], and they have been identified in various species. In rodents and humans one gene for cathelicidin is known. This homolog gene encodes for the antimicrobial peptide LL-37 in humans and CRAMP [9] and rCRAMP [10] in mice and rats, respectively.

Recent studies suggest that the expression of antimicrobial peptides, like human beta defensine 2 (HBD-2) is mediated through toll-like receptors 2 and 4 [11,12]. Furthermore it is indicated that the chemotactic G protein-coupled receptor formyl peptide receptor like-1 (FPRL1), expressed on both astrocytes and microglia, or the scavenger receptor MARCO (macrophage receptor with collagenous structure) play an essential role in the inflammatory response of host defense mechanisms. It has been shown that *Neisseria meningitidis *is a ligand of and mediates host defence against infection through MARCO [13,14]. Furthermore, MARCO is required for lung defence against pneumococcal pneumonia [15]. In addition a recent study showed an interaction between FPRL1 and MARCO in Aβ1-42-induced signal transduction in glial cells [16].

In this study we examined the time-dependent expression and co-localization of MARCO to glial cells in a model of experimental pneumococcal and meningococcal meningitis via fluorescence microscopy and using real-time RT-PCR studies of primary rat glia cells. We have been able to show a strong increase of MARCO in meningococcal meningitis co-localized to astrocytes. We investigated the function of FPRL1 and MARCO in the signal transduction of *Neisseria meningitidis *(NM) and *Streptococcus pneumoniae *(SP) by measuring extracellular-signal regulated kinase 1/2 (ERK 1/2) phosphorylation and cAMP levels in glial and transfected HEK cells. Receptors were inhibited by small interfering RNA and the consequences in NM- and SP-induced Camp (gene name for rodent cathelicidin) or proinflammatory cytokine interleukin 1β (IL-1β) expression and signal transduction were determined. Our results demonstrate the involvement of FPRL1 and MARCO in NM- and SP-mediated signalling. The results suggest that FPRL1 plays a pivotal role in NM-induced signal transduction in glial cells, and also show the capability of FPRL1 to expand its inflammatory ligand spectrum by interaction with the scavenger receptor MARCO.

## Methods

### Reagents

For the production of bacterial culture supernatants, bacteria were grown in stationary broth cultures for 20 h at 37°C until they had reached an optical density of 1.0. One ml of this culture was added to 9 ml of fresh medium and this was incubated for further 24 h in 75 cm^2 ^flasks without shaking. Brain heart infusion broth was used for the cultivation of *Streptococcus pneumoniae*, and thioglycolate broth supplemented with K1 and hemin was used for *Neisseria meningitidis*. Broth cultures of the test bacteria were centrifuged at 5000×g for 15 min, and the resulting supernatants were filter-sterilized. The supernatants were diluted 1:3 in EpiLife medium (Sigma, Germany) and used for stimulation experiments. Growth medium (diluted 1:100 or 1:200) served as the negative control to evaluate unspecific effects. Cells were challenged with supernatants from *Neisseria meningitidis *(ATCC 13077, type strain isolated from a fatal case of meningitis; 1:100) or *Streptococcus pneumoniae *(ATCC 6303; capsula type 3; 1:100) in serum and antibiotic-free DMEM.

FPRL1 antagonist WRW4 was purchased from Dr. P. Henklein (Charité, Berlin, Germany). The peptide was dissolved at 10 mM concentration in DMSO. Pertussin toxin (PTX) and formyl-methionyl-leucyl-proline (fMLF) were obtained from Sigma Chemical Company, Germany. pERK and ERK2 antibodies were obtained from Santa Cruz Biotechnology (CA, USA).

### Cell Culture

Isolated cerebral cortices and rostral mesencephali from 2-day old rats were stripped of the meninges, minced and dissociated enzymatically with trypsin from bovine pancreas (Sigma) in phosphate-buffered saline and 50 μg/mL DNase I (Roche Molecular Biochemicals, Indianapolis, IN, USA) for 30 min at 37°C and crushed mechanically with Pasteur pipettes. Astrocytes were prepared following the McCarthy and DeVellis method [17], which allows for the preparation of nearly pure cultures of astrocytes (> 97%) and cultivated in Dulbecco's modified Eagle's medium (DMEM) supplemented with 10% fetal calf serum (FCS). Suspended microglial cells were plated in 75 cm^2 ^cell culture flasks in microglial cell growth medium and harvested as described [18]. Prior to replating microglial cells for different assays, cell number and viability was estimated by trypan blue exclusion. This procedure increased the purity of the microglial preparation to > 98% with few astrocytes remaining. To test cell purity, cultures were stained with specific cell markers for astrocytes (glial fibrillary acidic protein (GFAP); astrocytes marker; Sigma) and microglia (OX42; microglia/macrophages marker; Sera-Lab, Leicestershire, UK). The production of primary rat meningeal cells was described previously [7].

HEK293 cells (American Type Culture Collection, Rockville, MD, USA) were subcultivated in DMEM supplemented with 10% FCS and 1% penicillin/streptomycin (Carl Roth, Karlsruhe, Germany). The transfection and selection of HEK293 cells expressing hMARCO, hFPRL1 or coexpressing hFPRL1-hMARCO was described previously [16].

### Astrocytes siRNA transfection

The astrocytes transfection was described previously [16]. Briefly, one day before transfection, 3 × 10^5^/well astrocytes were seeded in DMEM containing 10% FCS in 6-well-plates and transfected with Primefect^® ^(Lonza, Riverside, USA) transfecting agent containing control siRNA (25 nM) and siRNA for target proteins (25 nM), respectively according to the manufacturer's recommendation. Small interfering RNA (siRNA) duplexes corresponding to rat FPRL1 and MARCO cDNA sequences (GenBank accession number XM_001057934, XM_218012 and XM_001054109) or control siRNA were purchased from Qiagen, Valencia, CA, USA. Cells were cultured for an additional 96 h and analyzed for mRNA expression via SYBR green real time PCR.

### RNA isolation and real time RT-PCR

Total RNA was isolated using the peqGold Trifast reagent (peqlab, Erlangen, Germany) according to the manufacturer's protocol. RNA samples were reverse-transcribed by moloney murine leukemia virus (MMLV) reverse transcriptase (Fermentas, Burlington, Canada) and random hexamer primers (Invitrogen, USA). The cDNA products were used immediately for SYBR green real-time RT-PCR for Camp (rat), FPRL1 as well as MARCO and TaqMan real-time RT-PCR was used for CAMP (gene name for human cathelicidin) and IL-1β. Gene expression was monitored using the StepOne Plus apparatus (Applied Biosystems, USA) according to standard protocol [19]. Relative quantification was performed using the ΔCt method which results in ratios between target genes and a housekeeping reference genes (GAPDH or 18 s). The primer for Camp, FPRL1 and MARCO were manufactured by Qiagen (USA; QuantiTect Primer Assay). The specificity of the amplification reaction was determined with a melting curve analysis. We performed relative quantification of the signals normalizing the various gene signals with glycerinaldehyde-3-phosphate dehydrogenase (GAPDH) signal (forward primer: TCTACCCACGGCAAGTTCAAC; reverse primer: TCTCGCTCCTGGAAGATGGT; Eurofins MWG Operon, Germany) for SYBR Green real time RT-PCR, and with 18 s for TaqMan real time PCR.

### Western blotting

For western blot analysis of MAP kinases phosphorylation, glial or HEK293 cells were seeded in DMEM containing 10% FCS. Cells were harvested in a lysis buffer (50 mM Tris pH 7.5, 100 mM NaCl, 5 mM EDTA, 1% Triton, 2 mM sodium orthovanadate, 2.5 mM sodium pyrophosphate, 1 mM glycerol 2-phosphate, 1 mM phenylmethylsulfonylfluoride). Proteins (5 μg for pERK and ERK2) were resolved in SDS sample buffer, and a western blotting procedure was performed as previously described in detail [20]. Membranes were incubated with polyclonal primary antibodies against pERK1/2 overnight at 4°C and subsequent detection was performed with peroxidase-labeled secondary antibodies. Antibody binding was detected via enhanced chemiluminescence (Amersham Pharmacia Biotech, Essex, UK). The membranes were then stripped and re-probed with anti-ERK2 antibody as a loading control. The western blot bands were densitometrically evaluated with the program PC-BAS, the pERK-bands were adjusted with their respective ERK-bands and subsequently, the values were referred to control (= 100%).

### Determination of receptor activity by measuring cyclic AMP accumulation

1.5 · 10^5 ^astrocytes/well, or transfected HEK293 cells or 5 · 10^5 ^microglia/well were seeded in 22-mm 12-well dishes with DMEM containing 10% FCS and incubated overnight. The medium was removed and replaced by 0.5 ml of serum-free DMEM medium containing 10 μM forskolin (for astrocytes; Sigma) or 25 μM forskolin (for microglia or HEK293 cells) plus agonists. Different forskolin concentrations were used because of different cell sensitivities to forskolin-stimulated adenylate cyclase activity. The cells were incubated at 37°C for 15 min, and the reaction was terminated by removal of the culture medium and addition of 1 ml of ice-cold HCl/ethanol (1 N; 1 : 100, v/v). After centrifugation the supernatant was evaporated, residues were dissolved in Tris-EDTA (TE) buffer (50 mM Tris-EDTA, pH 7.5), and cAMP content was determined using a commercially available radioimmunoassay kit (Amersham Pharmacia Biotech).

### Infant rat model of experimental pneumococcal or meningococcal meningitis

An established model of pneumococcal meningitis in infant rats was used [21]. The animal studies were approved by the Animal Care and Experimentation Committee of the Canton of Bern, Switzerland. Wistar rats were infected on postnatal day 11 by direct intracisternal injection of 10 μl saline solution containing a defined inoculum of *Streptococcus pneumoniae *(serogroup 3) or *Neisseria meningitidis *with a 32-gauge needle in the cisterna magna. The animals were sacrificed at 12, 22 and 39 h for SP respectively 24 h for NM after infection (n = 3/time intervall). Uninfected control animals were injected with 10 μl of a sterile saline solution. To document existence of bacterial meningitis, CSF was obtained by puncturing the cisterna magna and cultured quantitatively. Animals were sacrificed, and perfused with 4% paraformaldehyde in PBS via the left cardiac ventricle for immunohistological studies.

### Fluorescence microscopy

Coronary brain sections (10 μm) from rats were cut on a cryostat, collected on Superfrost Slides (Merck, Braunschweig, Germany), air dried for 24 h and stored at −80°C. For immunofluorescence staining, sections were fixed with acetone (5 min), permeabilized with 0.1% Trition × in PBS for 10 min at room temperature, incubated with rabbit anti-MARCO (1:100; sc-68913, Santa Cruz, USA) and mouse anti-GFAP (astrocytes marker; ab10062, Abcam, Cambridge, UK) overnight at 4°C. Finally, the slices were incubated with donkey anti-rabbit AlexaFluor 488 (Molecular Probes, USA) or goat anti mouse Cy3 (Sigma, Germany) for 1 h at room temperature. Primary rat glial or different HEK293 cells were grown on glass coverslips. Cells were then exposed to NM or SP for 12 or 24 h at 37°C. After fixation with ice cold methanol and acetone for 5 min and 20 seconds, cells were blocked in 0.1 M Tris-HCl pH 7.5 containing 1,5% bovine serum albumin (BSA; Sigma Chemical Company, Germany) for 10 min. Coverlips were incubated at 4°C overnight with primary antibodies diluted in TRIS containing 1,5% BSA. The following primary antibodies were used for this study: rabbit anti-MARCO antibody (1:100; Santa Cruz, USA), rabbit anti-FPRL1 antibody (1:100; sc-18191, Santa Cruz, USA), goat anti-rCRAMP antibody (1:100; sc-34170, Santa Cruz, USA), and rabbit anti-LL-37 antibody (1:100; ab64892, Abcam, Cambridge, UK). Finally, the coverlips were incubated with donkey anti-rabbit AlexaFluor 488 or donkey anti-goat AlexaFluor 488 (all 1:250; Molecular Probes, USA) for 1 h at room temperature. Nuclear staining was performed with bisbenzimide (Sigma, Germany). Cells were digitally photographed using a Zeiss Axio Z1 Imager microscope (Zeiss, Göttingen, Germany).

### Statistical analysis

All experiments were performed at least in triplicate and the values represent mean ± SEM. The significance of the difference between test and control groups was analyzed using the ANOVA test, followed by the Bonferroni test. Data from the real-time RT-PCR, from densitometric quantification of western Blots, and from cAMP assay were analyzed using GraphPad Prism 5.0 software.

## Results

### Bacterial supernatants of Neisseria meningitidis induced MARCO expression in primary rat astrocytes and meningeal cells

**Figure 1:**
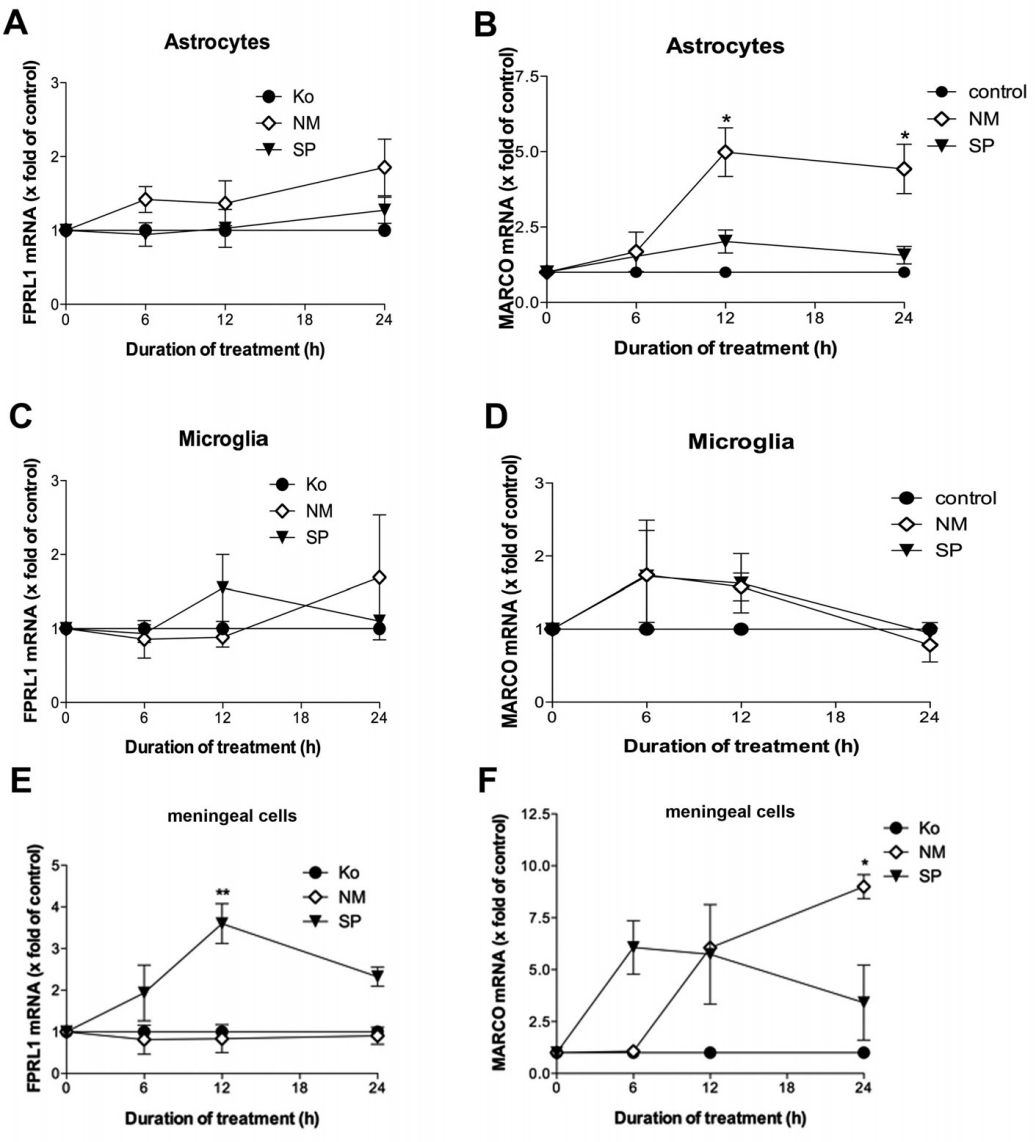
**Bacterial supernatants of *Neisseria meningitidis *induced MARCO expression in primary rat astrocytes and meningeal cells**. Astrocytes (A and B), microglia (C and D) and meningeal cells (E and F) were incubated with bacterial supernatants of Gram-positive bacteria *Streptococcus pneumoniae *(SP) or Gram-negative bacteria *Neisseria meningitidis *(NM) for 0, 6, 12 and 24 h. FPRL1 or MARCO mRNA expression was analyzed using SYBR green real-time RT-PCR and results were compared to the untreated sample. GAPDH (housekeeping gene) was used as an internal control. The data were assessed from three independent experiments in triplicate. An asterisk indicates a significant difference (**, p < 0.001; *, p < 0.05) compared to control as determined by ANOVA followed by the Bonferroni test.

In a first set of experiments we investigated the expression of both the FPRL1 and MARCO receptor by primary astrocytes, microglia and meningeal cells after stimulation with either NM or SP bacterial supernatants for 0, 6, 12, 24 h by real-time RT-PCR technique. As shown in Figure 1A and 1C, NM or SP does not induce a significant increase of FPRL1 expression in astrocytes as well as microglia. Only in meningeal cells, SP induced a significant increase of FPRL1 expression after 12 h of treatment (4 ± 0.5-fold increase in expression; Figure 1E). In astrocytes, a significant increase of MARCO expression by NM after 12 h of treatment was detected (5 ± 0.8-fold increase in expression; Figure 1B). For SP treatment, we could not detect an increase of MARCO expression. In microglia no significant changes of receptor expressions were observed after NM as well as SP treatment (Figure 1D). In meningeal cells, maximum MARCO expression was reached by NM after 24 h of treatment and by SP after 6 h of treatment (9 ± 0.6- and 6.1 ± 1.3-fold increase in expression, respectively; Figure 1F).

**Figure 2:**
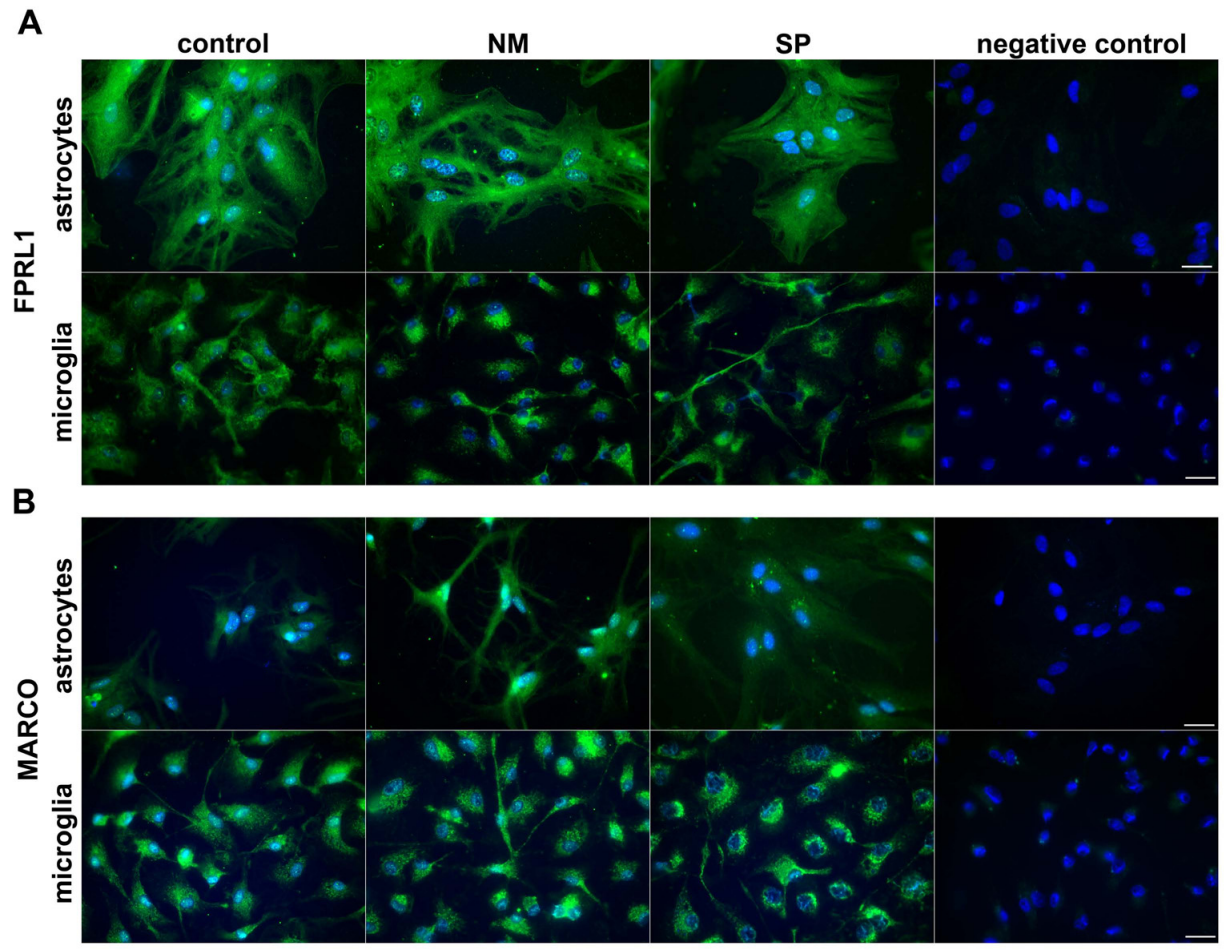
***Neisseria meningitides *bacterial supernatants induced an increase of MARCO immunoreactivity in astrocytes**. Primary rat astrocytes and microglia were fixed and labelled with anti-FPRL1 and anti-MARCO antibodies. FPRL1 (A) and MARCO (B) protein expression was examined by immunofluorescence microscopy. Bisbenzimide was used for nuclear counter-staining (blue). The figures show representative results from one of three independent experiments. Scale bar: 20 μm.

We then examined the FPRL1 and MARCO expression in primary rat astrocytes and microglia cells after treatment with NM and SP bacterial supernatants for 24 h using fluorescence microscopy. For FPRL1, both primary cells showed a clearly protein expression, but NM as well as SP could not induce an increased receptor expression (Figure 2A). For MARCO, we could detect an increase of protein expression in astrocytes after NM treatment, whereas SP induced no change of receptor expression in astrocytes as well as microglia (Figure 2B).

### Strong increased MARCO expression in astrocytes after NM infection in an infant rat model of meningitis

**Figure 3:**
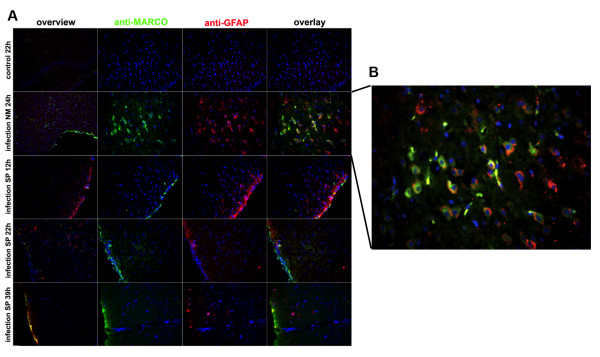
**Strongly increased MARCO expression in astrocytes after *Neisseria meningitides *infection in an infant rat model of meningitis**. Coronal brain sections from rats infected with *Neisseria meningitidis *(NM) or *Streptococcus pneumoniae *(SP) were fixed at defined time-points after infection and immunolabeled using anti-MARCO (green) and anti-glial fibrillary acidic protein (anti-GFAP) antibodies to identify astrocytes with nuclear counterstaining (blue). Sections were examined by double fluorescence microscopy. The figures show representative results from one of three independent experiments. Scale bar = 200 μm for overview and 20 μm for the other images. **(B) **Shows a detail of **(A)**. Please note that the detail shows a strong colocalization between GFAP and MARCO in the sub-cortical layer after NM infection.

In order to validate our previous *in vitro *findings *in vivo*, we used double fluorescence microscopy with receptor specific antibodies to examine the localization of MARCO and GFAP in astrocytes from infant rats with bacterial meningitis with NM or SP. Control rat brains showed a very low positive GFAP staining, since GFAP is a marker for activated astrocytes [22]. In correspondence with real-time RT-PCR and immunofluorescence results, MARCO expression shows a strong increase after 24 h of infection with NM as seen in Figure 3A, with visible co-localization of MARCO and GFAP in the sub-cortical area; Figure 3B. In addition the meninges showed an increase of MARCO expression after NM infection. Positive MARCO staining and MARCO/GFAP co-localization were also shown after 12, 22, 39 h of infection with SP, albeit weaker than in meningitis by NM (Figure 3A). After SP infection an increase of MARCO expression in meninges was also detected. Interestingly we could show an increase of MARCO expression after treatment with NM and SP in meningeal cells. For FPRL1 however, only SP induced increased expression (see Figure 1E and 1F).

### Inhibition of NM- as well as SP-induced Camp (rat) expression by FPRL1 antagonist WRW4 and G-protein inhibitor pertussis toxin in glial cells

**Figure 4:**
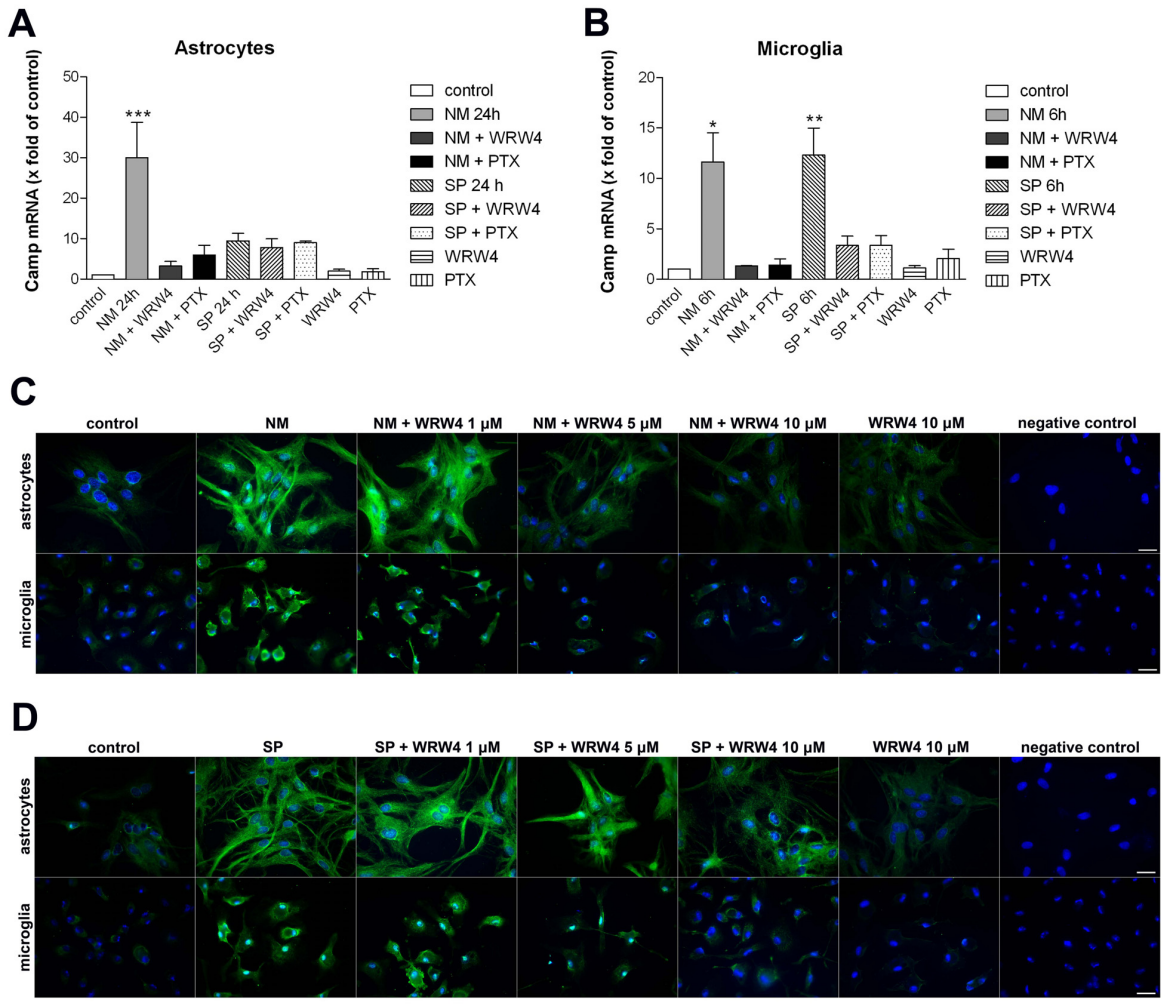
**Inhibition of *Neisseria meningitidis *as well as *Streptococcus pneumoniae*-induced Camp (rat) expression by the FPRL1 antagonist WRW4 and the G-protein inhibitor pertussis toxin in glial cells**. Bacterial supernatants from *Neisseria meningitidis *(NM) or *Streptococcus pneumoniae *(SP) were added to astrocytes **(A) **and microglia **(B) **with the addition of 200 ng/ml PTX (16 h preincubation) or 10 μM WRW4 (30 min preincubation) and with PTX (16 h preincubation) and WRW4 (30 min preincubation) alone to analyze the effect on Camp mRNA expression after 24 h (astrocytes; **a**) or 6 h (microglia; **b**) of treatment. The induction was analyzed and compared to an untreated sample (also with DMSO in equivalent amount). GAPDH (housekeeping gene) was used as an internal control. The data from three independent experiments performed in triplicate were assessed. An asterisk (*, p < 0.05; **, p < 0.01) indicates a significant difference between Camp expression after treatment and control (as determined by ANOVA followed by the Bonferroni test). Astrocytes or microglia were incubated with NM **(C) **or SP **(D) **with or without 1, 5 or 10 μM WRW4 (30 min preincubation) and WRW4 alone for 24 h or 12 h, respectively. Glial cells were fixed and labelled with anti-rCRAMP antibodies and protein expression was examined by immunofluorescence microscopy. Bisbenzimide was used for nuclear counter-staining (blue). The figures show representative results from one of three independent experiments. Scale bar: 20 μm.

Our previous results show a physical and functional interaction between FPRL1 and MARCO [16]. Furthermore we show an NM/SP-induced Camp (rat) expression in glial cells with a maximum at 24 h for astrocytes respectively 6 h for microglia [6]. Therefore, we investigated the possible involvement of FPRL1 in NM/SP-induced Camp expression in glial cells using real-time RT-PCR. As shown in Figure 4A, astrocytes were incubated with NM or SP with or without the FPRL1 antagonist WRW4 or the G-protein inhibitor pertussis toxin (PTX) for 24 h and Camp expression was determined. For microglia (Figure 4B), the cells were stimulated for 6 h. The results show that WRW4 and PTX strongly decreased NM-induced Camp expression (30.0 ± 8.8-fold increase) in astrocytes. For SP treatment, we could not detect a change of Camp expression after WRW4 or PTX co-stimulation, but also SP alone did not significantly induce Camp expression. In microglia, NM-induced Camp expression (11.7 ± 2.9-fold increase) was inhibited by WRW4 or PTX (Figure 4B). For SP treatment, WRW4 as well as PTX also decreased Camp expression.

To confirm the real-time RT-PCR results, we examined rCRAMP protein expression in primary rat astrocytes and microglia cells after treatment with NM and SP with different WRW4 concentrations using fluorescence microscopy. For NM, bacterial supernatants induced rCRAMP protein expression in glial cells after 24 h of treatment in astrocytes and 12 h of treatment in microglia (Figure 4C). NM-induced rCRAMP expression was strongly decreased by 5 and 10 μM WRW4 in astrocytes as well as microglia. Also, SP induced rCRAMP expression in astrocytes and microglia after 24 and 12 h of treatment, respectively (Figure 4D). In astrocytes, only 10 μM WRW4 showed an inhibition of SP-induced rCRAMP expression, whereas in microglia, 5 and 10 μM WRW4 decreased SP-induced rCRAMP expression. WRW4 alone could not induce rCRAMP expression in astrocytes or in microglia.

### Inhibition of NM- and SP-induced ERK1/2 phosphorylation and change of cAMP levels by WRW4 and PTX in glial cells

**Figure 5:**
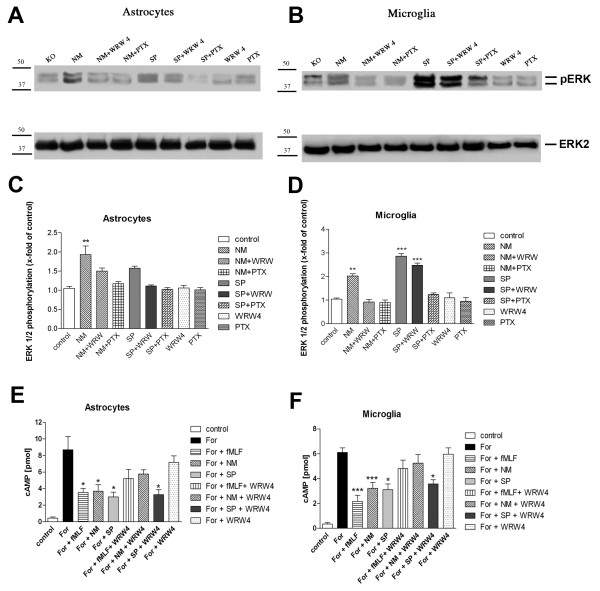
**Inhibition of *Neisseria meningitidis*- and *Streptococcus pneumoniae-*induced ERK1/2 phosphorylation, and changes of cAMP levels by WRW4 and PTX in glial cells**. For analysis of ERK1/2 phosphorylation, astrocytes **(A) **and microglia **(B) **were each treated with *Neisseria meningitidis *(NM) or *Streptococcus pneumoniae *(SP); with NM as well as SP for 5 min at 37°C with 200 ng/ml PTX (16 h preincubation) or 10 μM WRW4 (30 min preincubation); they were also treated with PTX (16 h preincubation) or WRW4 (30 min preincubation) alone as control. Cells were lysed, equal amounts of protein (5 μg) were dissolved in SDS sample buffer, and the levels of total ERK2 and phosphorylated ERK1/2 were determined via immunoblotting. The positions of phospho-ERK1/2 (pERK1/2) and total ERK2 (ERK2) along with those of the molecular mass markers (in kDa) are indicated on the right or left side, respectively. The values representing mean ± standard error of the mean (SEM) of phosphorylation levels derived from densitometric quantification of three independent experiments in astrocytes and microglia are indicated in **(C) **and **(D)**, respectively. An asterisk indicates a significant difference (*, p < 0.05; **, p < 0,01; ***, p < 0,001) compared to controls (also with DMSO in equivalent amount) as determined by one-way ANOVA and Bonferroni post-hoc tests. In order to analyse the inhibition of forskolin-stimulated adenylate cyclase activity, astrocytes **(E) **and microglia **(F) **were subjected to either 10 μM **(E) **or 25 μM **(F) **forskolin as well as to NM, SP or 1 μM fMLF and NM, SP or fMLF with 10 μM WRW4 (30 min preincubation) and to WRW4 alone for 15 min at 37°C. cAMP levels were determined as described above (see Methods). The values given represent mean ± SEM from four independent experiments. Asterisks indicate significant differences (*, p < 0.05; ***, p < 0,001) between forskolin plus agonists and forskolin alone as determined by one-way ANOVA and Bonferroni post-hoc tests.

To investigate the effect of FPRL1 on NM- as well as SP-induced glial cell activation we incubated primary rat astrocytes and microglia with NM or SP to determine ERK1/2 phosphorylation and changes of cAMP accumulation. As shown in Figure 5A to 5D, treatment with NM as well as SP resulted in increased phosphorylation of ERK1/2 in both astrocytes (NM: 1.9 ± 0.2; SP: 1.6 ± 0.1) and microglia (NM: 2.0 ± 0.1; SP: 2.9 ± 0.1). Therefore, as a next step, we tested the influence of NM as well as SP on changes in cAMP levels in glial cells. To determine whether NM and SP affected cAMP formation via G-protein receptor activity, cAMP production in glial cells was induced by forskolin treatment (8.7 ± 1.6 pmol cAMP). Interestingly, NM (2.9 ± 0.3 pmol cAMP) as well as SP (3.0 ± 0.6 pmol cAMP) induced a decrease in forskolin-induced cAMP accumulation in glial cells (Figure 5E and 5F). Apparently their activity is stimulated by an inhibiting G-protein. The FPRL1 agonist fMLF (2.5 ± 0.9 pmol cAMP) was used as a positive control. Next, we tested whether SP- or NM-induced ERK1/2 phosphorylation and changes in cAMP levels are mediated by inhibiting G-protein coupled receptor FPRL1. We used the FPRL1 antagonist WRW4 and pertussis toxin (PTX) as an inhibitor of inhibitory G-proteins. We first investigated the inhibition of ERK1/2 phosphorylation by the antagonists. As shown in Figure 5A and 5C, preincubation with WRW4 and PTX inhibited NM- as well as SP-induced ERK1/2 phosphorylation in astrocytes. In microglia, NM-induced ERK1/2 phosphorylation was decreased using WRW4 or PTX (Figure 5B and 5D). For SP, the induced ERK1/2 phosphorylation was inhibited by PTX, whereas WRW4 co-stimulation did not show a significant decrease. No changes could be detected in ERK1/2 phosphorylation with WRW4 and PTX alone. In addition to the assay mentioned above, the effects of antagonists on NM- or SP-induced cAMP formation was investigated. As shown in Figure 5E and 5F, fMLF and NM-mediated decreases in cAMP levels were inhibited by WRW4 in both astrocytes and microglia. For SP, in astrocytes as well as microglia, WRW4 had no effect on an SP-induced decrease of cAMP level. WRW4 alone did not alter forskolin-stimulated adenylate cyclase activity in glial cells.

### Inhibition of FPRL1 or MARCO expression in astrocytes by siRNA results in a decrease of NM- as well as SP-induced Camp as well as IL-1β expression and ERK1/2 phosphorylation

**Figure 6:**
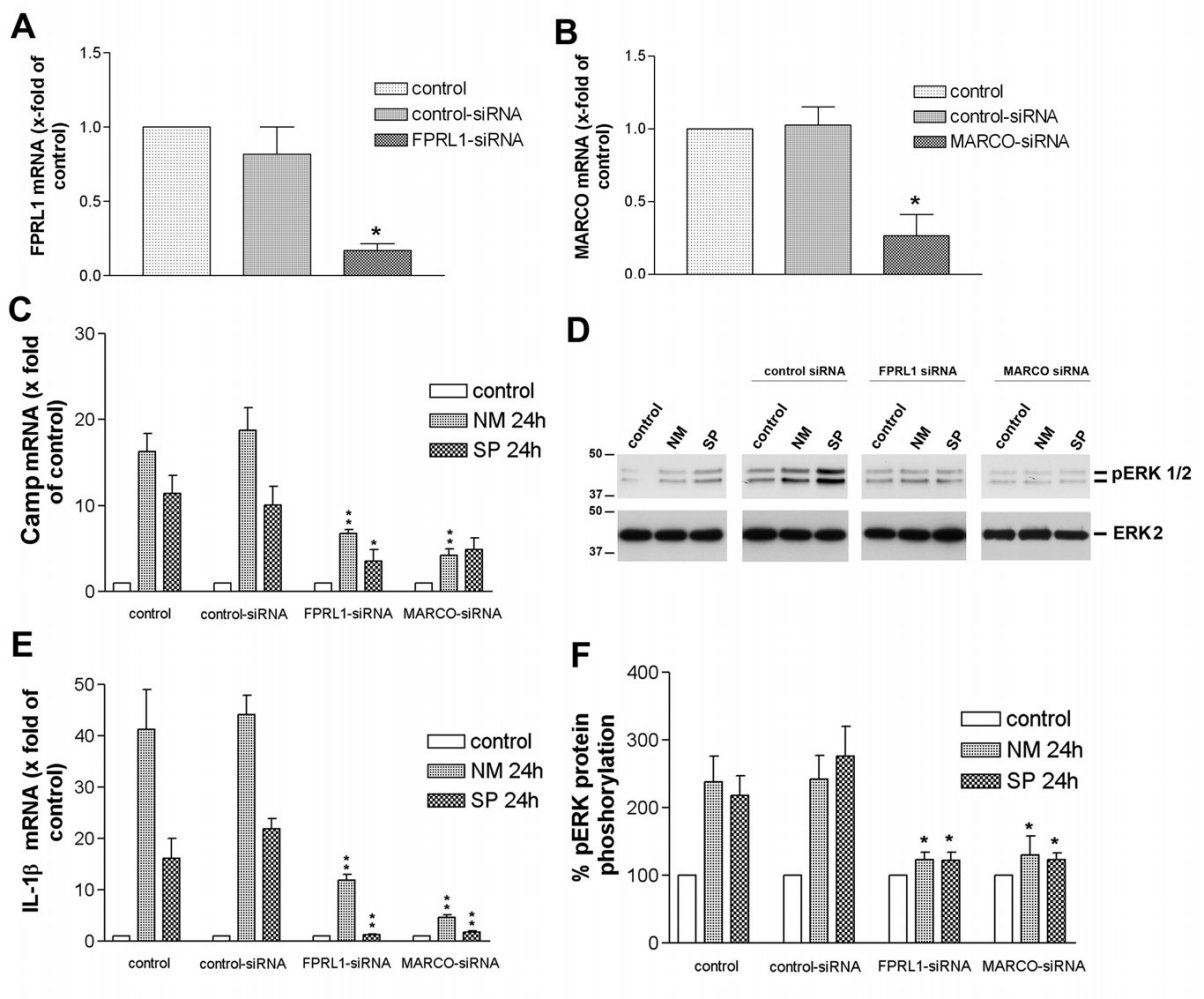
**Inhibition of FPRL1 or MARCO expression in astrocytes by siRNA resulted in a decrease of NM- as well as SP-induced Camp as well as IL-1β expression and ERK1/2 phosphorylation**. siRNA for FPRL1 and MARCO, as well as control siRNA, was transfected in astrocytes and down-regulation of FPRL1 **(A) **and MARCO **(B) **mRNA expression was analyzed 96 h later using SYBR green real time RT-PCR and compared to the untreated sample. GAPDH was used as an internal control (housekeeping gene). Data were assessed from three independent experiments each performed in triplicate. Asterisks indicate a significant difference (*, p < 0.05) compared to control siRNA (one-way ANOVA followed by the Bonferroni test). Transfected astrocytes were incubated 96 h, then incubated with SP or NM for 24 h and Camp **(C) **or IL-1β **(E) **mRNA expression was determined using SYBR green or TaqMan real time RT-PCR and compared to the untreated sample. GAPDH or 18 s was used as an internal control (housekeeping gene). Data were assessed from three independent experiments each performed in triplicate. Asterisks indicate a significant difference (*, p < 0.05; **, p < 0.001) compared to NM or SP stimulated astrocytes transfected with control siRNA (one-way ANOVA followed by the Bonferroni test). 96 h after transfection, **(D) **astrocytes were treated with NM or SP for 5 min at 37°C. Levels of total ERK2 and phosphorylated ERK1/2 were determined using immunoblotting. The mean ± SEM of the three independent experiments was evaluated by densitometric quantification **(F)**. Asterisks indicate significant difference (*p < 0.05) compared to control (one-way ANOVA followed by the Bonferroni test).

To determine whether FPRL1 as well as MARCO are essential for NM- as well as SP-induced Camp as well as cytokine expression and ERK1/2 phosphorylation, we prepared siRNA targeting rat FPRL1 and MARCO in astrocytes. Transfection of astrocytes with FPRL1 and MARCO siRNA, but not with control siRNA, resulted in a significant reduction in FPRL1 and MARCO mRNA levels 96 h after transfection as determined by SYBR green real time RT-PCR (Figure 6A and 6B). The transfection of primary rat microglia did not reach sufficient efficiency (data not shown). To investigate the effect of FPRL1 and MARCO for NM- as well as SP-induced Camp or IL-1β expression, astrocytes were transfected with FPRL1 or MARCO siRNA as described above. Ninety-six hours after transfection, Camp and IL-1β expression was determined using real-time RT-PCR. As shown in Figure 6C, treatment with NM as well as SP for 24 h resulted in a strong increase of Camp expression in astrocytes that had been transiently transfected with control siRNA and also in untransfected control cells. Transfection with FPRL1 but also MARCO siRNA significantly inhibited NM- as well as SP-induced Camp expression in astrocytes. Also the NM- and SP-induced proinflammatory cytokine IL-1β expression was significantly reduced by FPRL1 as well as MARCO siRNA inhibition (Figure 6E). The receptor activity was further determined by ERK1/2 phosphorylation. As shown in Figure 6D and 6F, treatment with NM and SP resulted in an intense phosphorylation of ERK1/2 in astrocytes that had been transiently transfected with control siRNA and also in untransfected control cells (about two- to threefold). Interestingly, transfection with FPRL1 as well as MARCO siRNA completely inhibited ERK1/2 phosphorylation induced by NM and SP.

### FPRL1 and MARCO mediate ERK1/2 phosphorylation and changes of cAMP levels in transfected HEK293 cells

**Figure 7:**
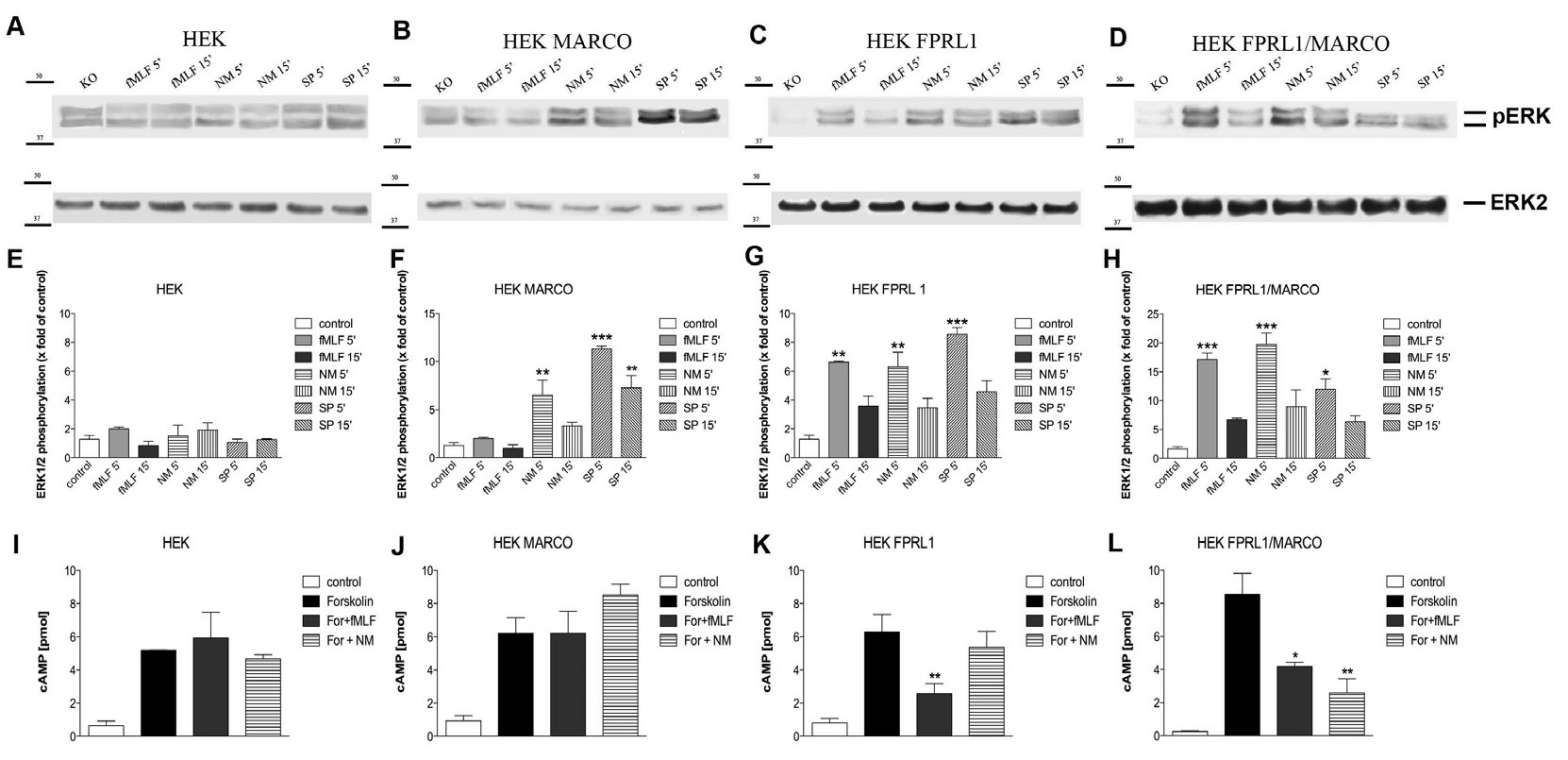
**FPRL1- and MARCO-mediated ERK1/2 phosphorylation and changes of cAMP levels in transfected HEK293 cells**. For analysis of ERK1/2 phosphorylation, untransfected **(A) **or hMARCO- **(B)**, hFPRL1- **(C)**, and hFPRL1/hMARCO- **(D) **expressing HEK293 cells were treated with *Neisseria meningitidis *(NM), *Streptococcus pneumoniae *(SP) or 1 μM fMLF for 5 and 15 min at 37°C. Cells were lysed, equal amounts of protein (5 μg) were dissolved by SDS sample buffer, and levels of total ERK2 and phosphorylated ERK1/2 were determined via immunoblotting. The positions of molecular mass markers are indicated on the left (in kDa). The mean ± SD of the three independent experiments were evaluated by densitometric quantification normalized to ERK2 expression **(E to H)**. Asterisks indicate a significant difference (*, p < 0.05; **, p < 0,01; ***, p < 0,001) compared to control (one-way ANOVA followed by the Bonferroni test). For analysis of inhibition of forskolin-stimulated adenylate cyclase activity, untransfected **(I) **or hMARCO- **(J)**, hFPRL1- **(K)**, and hFPRL1/hMARCO- **(L) **expressing HEK293 cells were subjected to 25 μM forskolin as well as to NM or 1 μM fMLF for 15 min at 37°C. cAMP levels were determined as described above (see Methods). The values represent mean ± SEM from four independent experiments. Asterisks indicate a significant difference (*, p < 0.05; **, p < 0,01) between forskolin plus agonists and forskolin alone, as determined via one-way ANOVA followed by the Bonferroni test.

In an effort to further evaluate the effect of FPRL1 and MARCO in NM/SP- and fMLF-induced signal transduction we used transfected HEK293 cells. Either hMARCO- or hFPRL1-expressing, or hFPRL1 and hMARCO co-expressing cells were generated and ERK 1/2 phosphorylation was analyzed after treatment with NM/SP and fMLF for 5 and 15 minutes. The results of the western blot were confirmed by densitometric quantification. In untransfected cells no treatment led to an increase in ERK 1/2 phosphorylation (Figure 7A and 7E). In hMARCO-transfected cells, a 5-minute treatment with NM (6.5 ± 1.5 fold increase) and SP (11.3 ± 0.3 fold increase) induced a significant increase in ERK1/2 phosphorylation (Figure 7B and 7F). As shown in Figure 7C and 7G, hFRPL1-transfected cells also showed this increase in phosphorylation, but here the treatment with the FPRL1 agonist fMLF (6.6 ± 0.1 fold increase) also led to increased phosphorylation after 5 minutes. Finally, in hFPRL1 and hMARCO co-expressing cells a 5-minute stimulation with all three substances significantly raised the level of ERK phosphorylation (fMLF: 17.1 ± 1.1; NM: 19.7 ± 2; SP: 12 ± 1.8; Figure 7D and 7H). It should also be mentioned that the increased phosphorylation after fMLF and NM treatment in the co-expressing cells was also significantly higher than that of cells transfected with only one of the receptors. The above results are reflected in a change in forskolin-induced adenylate cyclase activity. In untransfected, or just hMARCO-transfected, cells no change in cAMP concentration was detected after treatment with fMLF and NM (Figure 7I and 7J). In hFPRL1-transfected cells only the stimulation with fMLF was able to reduce the cAMP level (2.6 ± 0.6 pmol; Figure 7K). Solely the FPRL1 and MARCO co-expressing cells showed a decrease in adenylate cyclase activity after treatment with either fMLF (4.3 ± 0.2 pmol) or NM (2.6 ± 0.9 pmol; Figure 7L).

### Increased CAMP/LL37 expression in transfected HEK293 cells

**Figure 8:**
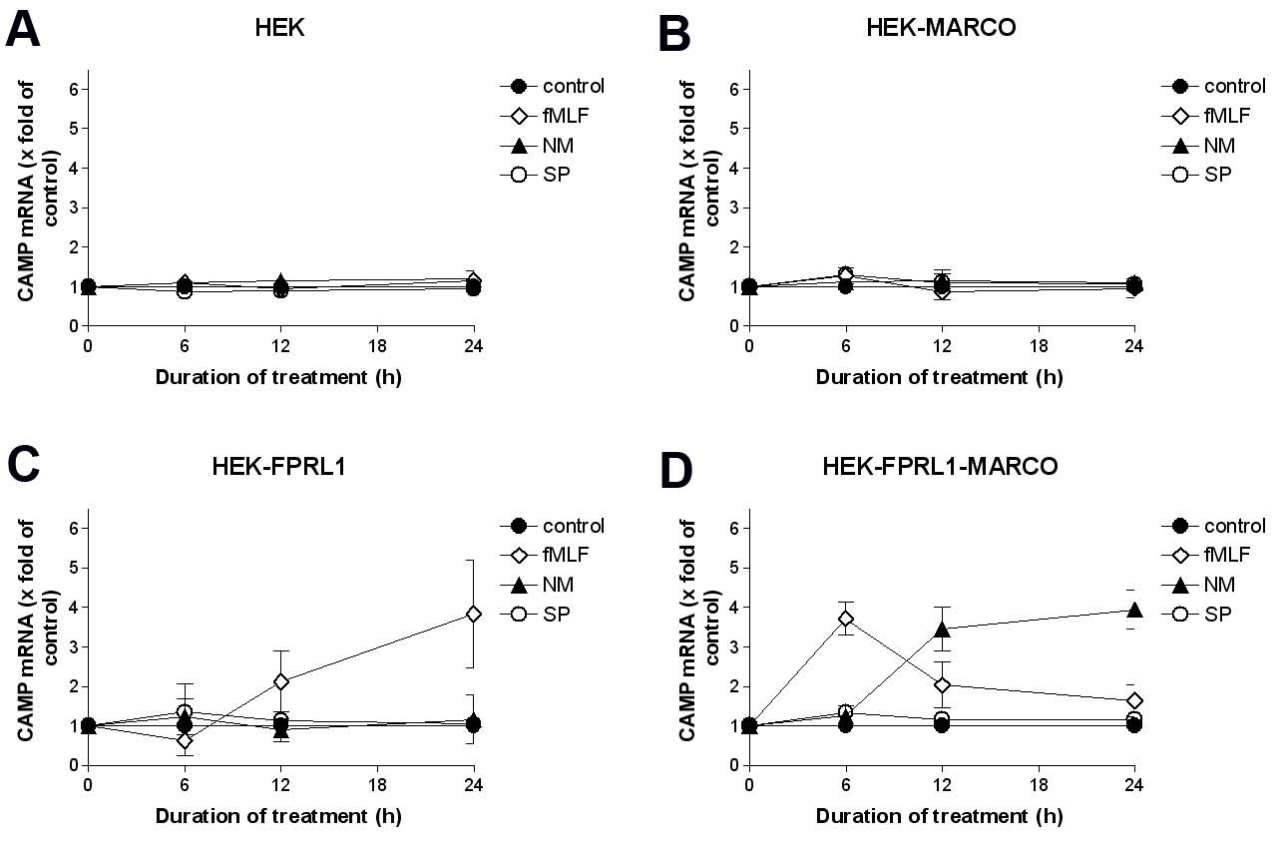
**Increased CAMP (human) expression in transfected HEK293 cells**. For analysis of CAMP mRNA expression, untransfected **(A) **or hMARCO- **(B)**, hFPRL1- **(C)**, and hFPRL1/hMARCO- **(D) **expressing HEK293 cells were treated with *Neisseria meningitidis *(NM), *Streptococcus pneumoniae *(SP) or 1 μM fMLF for 0, 6, 12 and 24 h. mRNA expression was analyzed using TaqMan real-time RT-PCR and results were compared to the untreated sample. 18 s (housekeeping gene) was used as an internal control. The data was assessed from three independent experiments.

In an additional set of experiments we investigated the effect of fMLF, NM and SP treatment for the human cathelicidin CAMP (LL-37) in transfected HEK293 cells mentioned above. After 0, 6, 12 or 24 h treatment with fMLF, NM and SP CAMP expression as analyzed by using real-time RT-PCR. As shown in Figure 8A to 8C, in untransfected and cells transfected only, with either hFPRL1 or hMARCO, stimulation with either substance did not change the expression of CAMP mRNA. Only in hFPRL1 and hMARCO co-transfected cells (Figure 8D) did the treatment with fMLF show a maximum increase of CAMP mRNA expression after 6 h (4-fold increase). NM induced a maximum induction of CAMP expression at 24 h of treatment (3.9-fold increase). SP did not alter CAMP expression in hFPRL1-hMARCO-expressing HEK293 cells.

**Figure 9:**
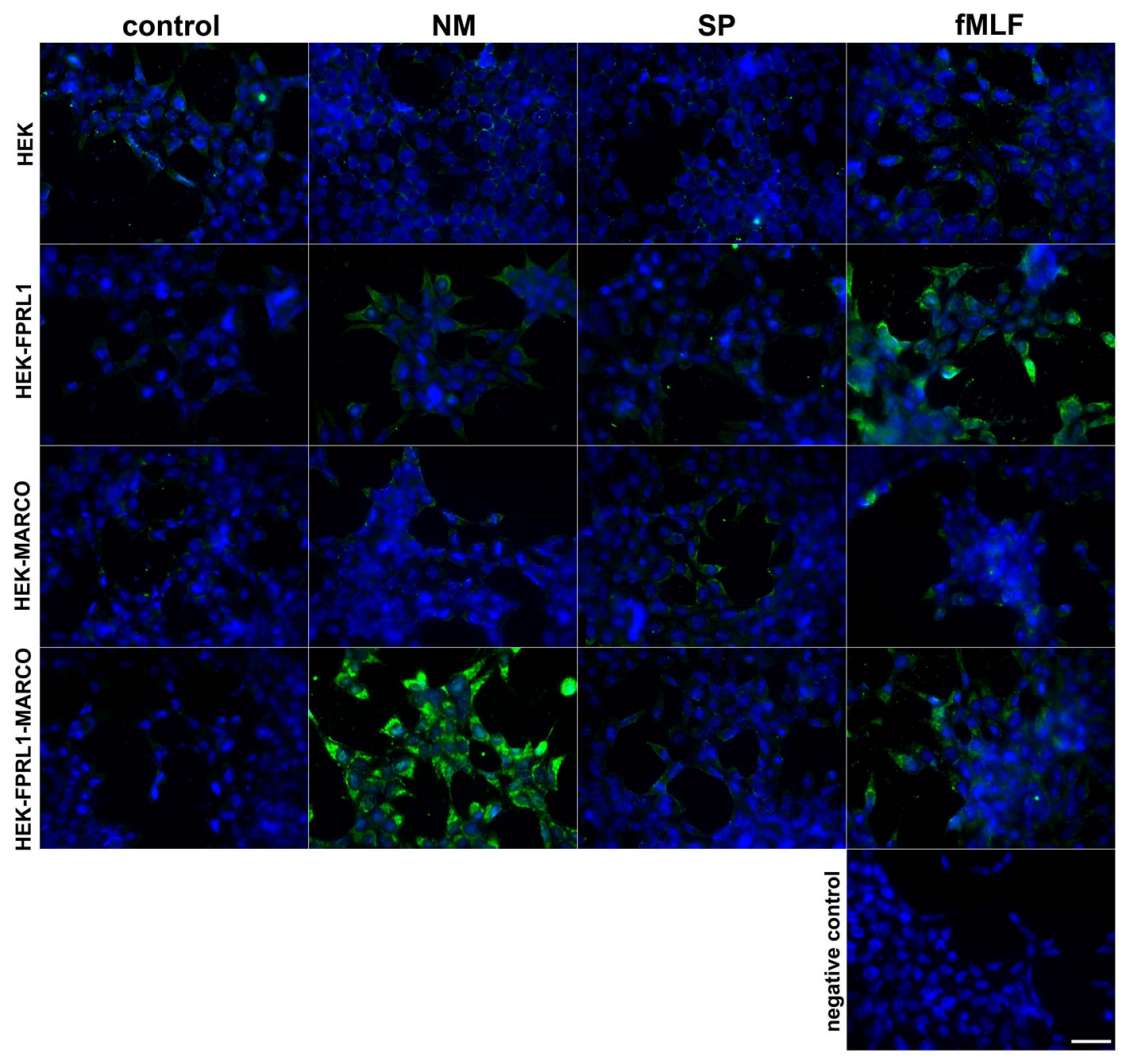
**Increased LL-37 protein expression in transfected HEK293 cells**. Untransfected or hFPRL1-, hMARCO- or hFPRL1/hMARCO-expressing HEK293 cells were incubated with NM or SP for 24 h. Cells were fixed and labelled with anti-LL-37 antibodies and protein expression was examined by immunofluorescence microscopy. Bisbenzimide was used for nuclear counter-staining (blue). The figures show representative results from one of three independent experiments. Scale bar: 20 μm.

To confirm the real-time RT-PCR results, we examined LL-37 protein expression in transfected HEK293 cells after treatment with NM, SP and fMLF for 24 h using fluorescence microscopy. As shown in Figure 9 in untransfected and in HEK293 cells transfected only with hMARCO, stimulation with either substance did not change the expression of LL-37 protein. In hFPRL1-transfected cells, fMLF induced a strong, and NM a weak, LL-37 protein expression, whereas in hFPRL1 and hMARCO co-transfected cells treatment with NM and fMLF showed a clear increase of LL-37 expression.

## Discussion

Our present study shows a functional influence of the FPRL1 and MARCO receptors in NM- and SP-induced signal transduction and expression of the antimicrobial peptide rCRAMP in glial cells. In previous investigations we were able to show an increased expression of LL-37/rCRAMP in bacterial meningitis localized to astrocytes and microglia [6], as well as a functional interaction between the FPRL1 and MARCO receptors in neurodegenerative disease signalling [16]. Now, we demonstrate differences between NM- and SP-induced glial cell activation. The activation of different signal transduction pathways (ERK1/2 and cAMP level measurement) and increased Camp (rat) and proinflammatory cytokine IL-1β expression in primary rat glia cells by NM and SP is dependent on the FPRL1 and MARCO receptor, as verified by antagonist treatment, receptor inhibition by siRNA and using transfected HEK293 cells.

Using an infant rat model of experimental pneumococcal and meningococcal meningitis and a primary glia cell culture, our results show a strong increase of MARCO expression after NM infection in astrocytes but not in microglia. FPRL1 expression in astrocytes as well as microglia was not changed (Figure 1 and 2), whereas SP induced an increase of FPRL1 expression in meningeal cells. MARCO expression in meningeal cells was increased by NM as well as by SP (Figure 1). For glial cells, we have previously been able to show a basal FPRL1 expression in astrocytes and an even stronger one in microglia [20]. For MARCO expression, Alarcon et al. [23] could show an equally strong endogen expression in astrocytes as well as microglia. As shown by Mukhopadhyay et al. [14] and Plüddemann et al. [24] NM is an important ligand for MARCO. MARCO is involved in defence against pneumococcal pneumonia [15]. This receptor mediates immunological responses in combination with other receptors, like Toll-like receptors and other scavenger receptors on macrophages. The glial cell reactions thus depend on the kind of the bacterium and on the expressed receptor pattern. However, in astrocytes, challenge with NM significantly increased MARCO expression, thus enhancing these cells' ability to react to and defend against Neisseria infection. Possibly, meningeal cells act as antigen-presenting cells in this compartment for glial or invading immune cells [25].

Differences between astrocytes and microglia also showed up regarding the NM/SP-induced signal transduction in glial cells (Figure 5). NM-induced ERK1/2 phosphorylation and changes of cAMP level were clearly inhibited by the FPRL1 antagonist WRW4, whereas for SP, the induced signal transduction was not decreased by WRW4. This is possibly based on differences between formyl peptides from Gram-positive and/or Gram-negative bacteria. In Gram-negative bacteria *Escherichia coli *culture supernatants, the chemotactic peptide fMLF is a major chemoattractant [26]. On the other hand, different receptors possibly play a more important role in SP-induced signal transduction in glial cells. It has been shown that pneumococcal infection of the central nervous system (CNS) depends on TLR2 and TLR4 [27]. In addition, an involvement of the pattern recognition receptor nucleotide-binding oligomerization domain-2 (NOD2) in the inflammatory response of murine glia to SP could be shown [28]. Furthermore, our results show that SP-induced ERK1/2 phosphorylation in microglia is PTX-sensitive. For ERK1/2 phosphorylation and changes of cAMP levels in glial cells, thus, at least one unknown G-protein coupled receptor seems to be involved (Figure 5). However, transfection of astrocytes with FPRL1 as well as MARCO siRNA reduced NM- and SP-induced ERK1/2 phosphorlylation (Figure 6). The results with transfected HEK293 cells show that in cells only transfected with hFPRL1 or hMARCO, SP and NM induce ERK1/2 phosphorylation (Figure 5). Interestingly, in HEK293 cells co-transfected with hFPRL1 and hMARCO, ERK1/2 phosphorylation was stronger than in other transfected cells after NM or SP treatment. Taken together, the data suggest a collaboration between FPRL1 and MARCO triggering cellular responses after NM or SP infection. While the change of cAMP level after NM treatment is possibly mediated by FPRL1, both receptors are involved in NM- and SP-induced ERK1/2 phosphorylation and have synergistic effects for ERK1/2 phosphorylation after NM or SP treatment.

In addition, the increased Camp expression by NM in the glial cells seems to be mediated generally by FPRL1 (Figure 4). However, binding of MARCO is needed because, considering our results with siRNA and in HEK293 cells, CAMP/LL-37 expression is linked to the presence of FPRL1 and MARCO (Figure 6 and 8). The involvement of a different receptor spectrum in SP-induced Camp expression can be assumed even more, considering the CAMP expression in transfected HEK293 cells. Here SP was not able to show any effect with only the FPRL1 and MARCO receptors present (Figure 8 and 9); but in astrocytes FPRL1 and MARCO siRNA reduced SP-induced Camp expression (Figure 6). Possible other receptor candidates in NM and SP signalling and Camp induction TLRs have to be mentioned [29]. This difference in expression could also explain the observed Camp expression in glia cells. CRAMP expression is most likely an important part of the glial cell-mediated immune response in bacterial meningitis. CRAMP KO mice show a clearly higher susceptibility for meningococcal infection than do WT mice [30]. Cruse et al. could show that human lung mast cell-released LL-37 is antimicrobial against SP [31]. Our previous studies demonstrate cytokine releasing and antimicrobial effects of rCRAMP/LL-37 in a meningitis setting [7]. In addition our results have demonstrated an rCRAMP-mediated glial cell activation, which accompanies an increase of neurotrophic factor expression by glial cells, so rCRAMP, apart from the immune response in the CNS, is also involved in neuroprotection [32]. Further investigations should clarify to what extent differences exist between the various meningitis strains. These findings could also offer an explanation for clinical differences concerning neurological sequelae of NM and SP [33,34].

In conclusion, our results show the importance of the FPRL1 and MARCO receptors in host defence against NM and SP, and show a cooperative signal transduction-enhancing effect of the MARCO receptor. Our results document important differences between the meningitis-causing bacterial pathogens NM and SP regarding glial cell activation. Future investigations could help develop more specific approaches against infections of the CNS.

## Abbreviations

The abbreviations used are: rCRAMP: rat cathelin-related antimicrobial peptide; LL-37: human cathelicidin: Camp: gene name for rodent cathelicidin; CAMP: gene name for human cathelicidin; ERK: extracellular signal-regulated kinases; DMEM: Dulbecco's modified Eagle's medium; FCS: fetal calf serum; FPRL1: formyl peptide receptor like-1; GFAP: Glial fibrillary acidic protein; PTX: pertussis toxin; NM: *Neisseria meningitides*; SP: *Streptococcus pneumoniae*; MARCO: Macrophage receptor with collagenous structure

## Competing interests

The authors declare that they have no competing interests.

## Authors' contributions

BJB and LOB designed and performed experiments, and drafted the manuscript. SLL performed the infant rat model of experimental pneumococcal or meningococcal meningitis. AS helped to accomplish experiments. RP, RL, SJ, CJW, DV and TP co-conceived of the study, participated in its design and coordination, and helped draft the manuscript. All authors have read and approved the final version of this manuscript.
